# A rare case of massive intrapartum hemorrhage followed by inner myometrial laceration during a vaginal delivery: A case report

**DOI:** 10.1002/ccr3.8373

**Published:** 2024-01-02

**Authors:** Bahareh Khakifirooz, Arian Shojaei, Amirhossein Hajialigol

**Affiliations:** ^1^ Department of Obstetrics and Gynecologists, School of Medicine, Kamali Hospital Alborz University of Medical Sciences Karaj Iran; ^2^ Kamali Hospital Alborz Iran; ^3^ Alborz Office of Universal Scientific Education and Research Network (USERN) Alborz University of Medical Sciences Karaj Iran

**Keywords:** increased intrauterine pressure, inner myometrial laceration, intrapartum hemorrhage, labor

## Abstract

**Key Clinical Message:**

Considering the laceration of the inner layer of the myometrium as an important and controllable cause of bleeding during childbirth can lead to saving the mother's life.

**Abstract:**

Laceration of the inner layer of the myometrium can cause massive bleeding during and after childbirth, which can lead to the death of the mother if it is not diagnosed in time.we presented a rare case of massive intrapartum bleeding following myometrial laceration that diagnosed correctly and the patient survived with in‐time treatments. The patient was a 26‐year‐old woman who was under observation for term pregnancy and complaint of rupture of membranes (ROM) and vaginal bleeding. Following the spontaneous course of labor and without receiving oxytocin, during the normal course of labor, she was with an estimated total blood loss of 750 mL bleeding, which despite the normal fetal heart rate and with the mother's indication for cesarean section, was transferred to the operating room and underwent cesarean section. During the cesarean section, the amniotic fluid was clear, after the removal of the placenta, severe and clear bleeding was flowing from the posterior wall of the uterus, which was caused by the laceration of the inner layer of the myometrium in the posterior wall of the lower segment of the uterus. The myometrial laceration was repaired with absorbable continuous locked sutures and hemostasis was established, and then the patient used uterotonic drugs, and after monitoring, the patient was discharged from the hospital in good condition.

## Graphical abstract

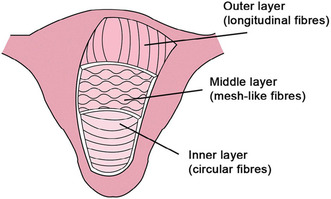

## 1 INTRODUCTION

Bleeding during labor is considered one of the obstetric emergencies, which if we miss the diagnosis can cause the death of the mother and the death of the fetus. The most important causes of bleeding during childbirth are maternal coagulation disorders, vaginal and cervical wall laceration, uterine laceration, placental abruption, and placenta previa.^1^

Rupture of the uterus, like the rupture of any internal organ (spleen, liver, etc.), is a life‐threatening situation for the mother and the fetus.^2^ Most of the ruptures of the uterus occur in people with a history of uterine scar (cesarean section, myomectomy). However, rare cases of uterine rupture without scars have been reported, which have been associated with increased mortality compared to uterine rupture with scars.^3^ The prevalence of uterine rupture without a history of scarring is reported about 0.7 per ten thousand births.^4^

These lacerations can be caused by trauma, connective tissue disorders like marfan syndrome, obstetric maneuvers (internal and external rotation, uterine fundal pressure), old age of the mother, multiparity, multiple births, twin pregnancies, uterus abnormalities, and uterotonic drugs.^1^, ^5^

Uterine fundal pressure or Kristeller maneuver is performed to accelerate the exit of the fetus in the second stage of labor, although, in various studies, the benefits of this maneuver have not been fully determined. This maneuver is performed to shorten the second stage of labor when the heart rate of the fetus drops or the mother's fatigue, which can increase intrauterine pressure. However, this maneuver plays a role in uterine rupture along with other risk factors.^6^, ^7^

In this case report, we presented a rare case of bleeding during delivery following the laceration of the myometrium layer that we considered connective tissue disorders like marfan syndrome as an etiology of the laceration.

**FIGURE 1:**
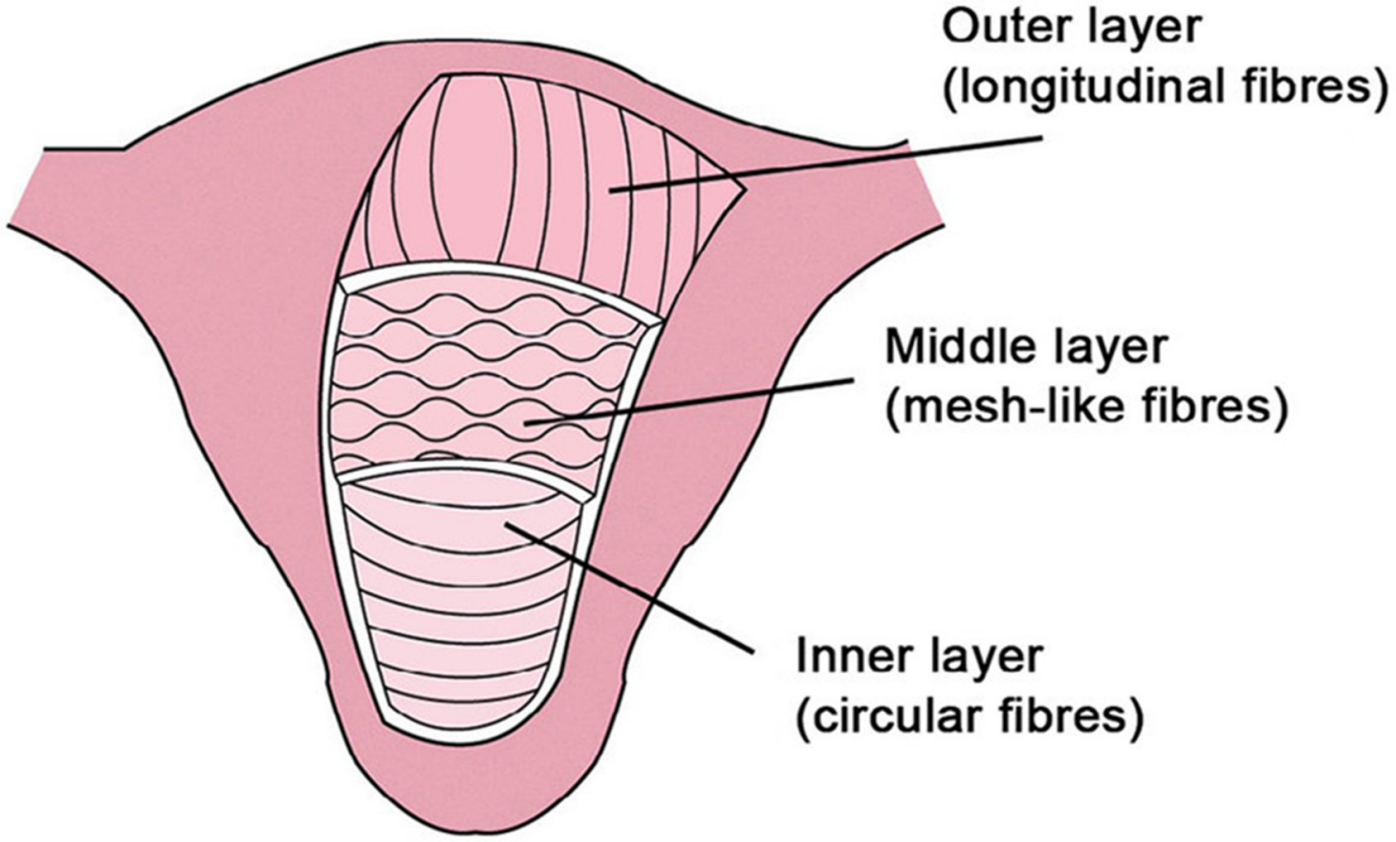
Uterine layers. Inner circular layer, middle “mesh‐like” layer, and outer longitudinal layer.^8^

## 2 CASE PRESENTATION

Our case was a 26‐year‐old primigravida woman without any underlying disease gestational age of her was 38 weeks and she has been referred to Kamali University Hospital in Alborz at 2 a.m. in January 2023.

Mother's vital signs were stable and there were no tachycardia or hypotension. The Complete blood count test, PTT, PT, liver, urea, and creatinine tests were all normal. After cephalic examination and auscultation of the fetal heart, she was admitted to the labor ward in early stage of labor with vaginal bloody rupture sac and her cervical dilatation was 3 cm with sac raptured bloody and 30% effacement and ‐3 station was reported.

The fetal heart rate monitoring and tocometry were performed at 5:30 a.m., and the NST was completely reassurance and reactive.

The contractions were appropriate and there was no uterine tachysystole, but the bleeding was still +2 and clear. In the reexamination after 5:45 a.m., cervical dilatation was 3 cm with sac raptured bloody and 60% effacement and ‐3 station. Vaginal bleeding was continuous despite reactive NST and no evidence of uterine tachysystole.

To control the course of labor, the mother and the fetus were under continuous monitoring after 6:40 a.m. Fetus examination was 5 cm of cervical dilatation, 75% effacement with cephalic presentation, and sac raptured bloody. She was candidate for emergency cesarean with maternal indication due to severe and continuous vaginal bleeding, so we took her to the operating room at 7:00 a.m, and at 7:25 a.m. she underwent spinal anesthesia, and at 7:30 a.m. a Kerr hysterotomy (transvaginal laparoscopic surgery (TLS)) was performed. A cephalic baby was born with an APGAR score of 9 out of 10 in the first minute and a weight of 2800 grams. The placenta was removed with a uterine fundal position. After the removal of the placenta, there was no evidence of placental abruption, there was no evidence of placental adhesion, but at the same time, there was heavy and clear bleeding from the posterior wall of the uterus. We distinguished the patient's laceration from the laceration due to medical maneuver that a cesarean incision was made in the anterior wall of the lower part of the uterus but the laceration created in the posterior wall of the lower part of the uterus was exactly behind the head of the fetus inside the uterus (Figure 1).

Due to the accurate diagnosis of the laceration of the inner layer of myometrium, the patient had stable hemodynamics during and after the surgery and did not need blood transfusion the estimated amount of the bleeding was 750 mL during the delivery. Hemoglobin level of the patient was in normal range before the surgery but during the delivery it started to drop and 12 h after the surgery it was 9.5 (Table 1) but after the diagnosis and management of the laceration, it back to normal range after 48 h.

**TABLE 1:** The course of the patient's hemoglobin level.

	Before surgery	6 h after surgery	12 h later
Hemoglobin level	12.8	10.1	9.5

During the examination, there was a 4 cm longitudinal transverse laceration in the inner layer of the myometrium in the posterior wall of the lower uterine segment. The outer layer of the myometrium and uterine serosa were completely healthy and intact, so we proceeded to repair the laceration of the inner layer of the myometrium during muscle repair, due to unrecognizable direction of the muscle fibers we took all of layers. Laceration was repaired with absorbable continuous locked sutures and hemostasis was established. After ensuring that hemostasis was stable, the surgery was finished and then the patient was delivered to the recovery room in good condition and discharged from surgery ward after 48 h of surgery with good general condition and no problems, also there were no problems in the examination 3 months after the surgery.

## 3 DISCUSSION

Intrapartum hemorrhage is one of the common causes of maternal morbidity which should be diagnosed and treated effectively.^8^ Laceration of the inner layer of the myometrium is a rare but one of the most important causes of intrapartum hemorrhage that should be considered in cases after ruled out other common causes^9^ Different causes for uterine rupture have been reported, and all of them have a common physiopathology, during normal vaginal delivery, uterine contractions, and intrauterine pressure increase, which increases the stress on the uterine walls. Based on the clinical findings of our patient and the knowledge from the previous articles, we considered connective tissue disorders like Marfan syndrome as an etiology of our patient's laceration.^5^, ^9^

According to the laws of physics (Pascal's law), the intrauterine pressure is distributed uniformly, but sometimes due to the position of the fetus and the accumulation of amniotic fluid around the fetus, the intrauterine pressure is unevenly distributed on the walls this distribution, causes increased stress on some parts of the uterus.^3^ Papers that reported myometrial laceration were not so many but the most important ones are below:

- The first report of myometrial laceration was by Hayashi et al. in 2000. Overally 37 cases were enrolled in this study, in that three cases of postpartum hemorrhage occurred due to myometrial lacerations. Hence, they assumed that an abnormal increase in intrauterine pressure during delivery that happens due to uterine contractions may impose high levels of pressure on the cervix and an inner myometrial laceration happens. They suggested that prescription of uterotonic compounds with appropriate doses and time intervals is necessary to prevent uterine laceration and excessive uterine contractions.^5^
- Kaplanglou et al., in 2016, studied four patients that they had normal vaginal delivery but with massive uncontrollable hemorrhage. They performed the emergency laparotomy and detected inner myometrial lacerations. The myometrial lacerations were sutured as a primary management; however, they did hysterectomy for two of these patients due to uncontrollable hemorrhage. They concluded that an inner myometrial laceration should be controlled by suturing the laceration. Considering the age of the patient and her parity, hysterectomy can be conducted when we have uncontrolled hemorrhage.^10^
- In the two cases reported by Malhotra et al. (2012) and Abu‐Rustum et al. (2006), both patients who had severe bleeding after natural vaginal delivery and were resistant to treatment, unlike the patient in the present report, were multiparous but the age of the mothers was 25–31 years old, while the case reported by Conrad et al. (2015) was 17 years old and was the primigravida.^3^, ^11^, ^12^
- In the study of Malhotra et al. (2012), pregnancy termination was done without induction, as in the case of the present study, while in the study of Abu‐Rustum et al. (2006) induction was done.^11^, ^12^ In all the reported cases, as in the case of present study, the term pregnancy and the weight of the babies were between 2700 and 3700, and there was no history of systemic disease in any of the cases.
- In the present patient, as in other reported cases, laparotomy was performed due to massive vaginal bleeding.^11^, ^12^
- In these cases, like the present patient, there was a rupture of the inner layer of the myometrium in the posterior wall of the uterus, with the difference that due to the diagnosis during the operation and its repair, the bleeding was controlled and there was no need for a hysterectomy. While the patients due to absence examining the internal parts of the uterus, myometrial rupture was not detected and finally, a hysterectomy was performed.
- In the study reported by Malhotra et al. (2012), the patient died in the ICU 2 days after the laparotomy, while in the study by Abu‐Rustum et al. (2006), like our study, the patient recovered completely after the repair of the inner layer of the myometrium and supportive measures. Faster protective measures and early laparotomy and diagnosis and repair of the rupture of the inner layer of myometrium saved the patient from death.

In summary it is very important to consider the rupture of the inner layer of myometrium in women with intrapartum or postpartum hemorrhage, especially in cases that do not respond to protective measures and medical treatments. The quick treatment of this condition (through the incision on the uterus and examination of the myometrium and its repair) can lead to faster control of bleeding and prevention of severe and maternal complications.

## AUTHOR CONTRIBUTIONS

**Bahareh Khakifirooz:** Data curation; investigation; supervision; writing – review and editing. **Arian Shojaei:** Data curation; investigation; validation. **Amirhossein Hajialigol:** Investigation; validation; visualization; writing – original draft.

## FUNDING INFORMATION

No funding was utilized for this manuscript.

## CONFLICT OF INTEREST STATEMENT

The authors declare that they have no competing interests.

## ETHICS APPROVAL AND CONSENT TO PARTICIPATE

Our study is a case report without any intervention on the patient, and informed consent has been obtained for publication.

## CONSENT

Written informed consent was obtained from the patient to publish this report in accordance with the journal's patient consent policy.

## References

[1] Dahlke JD , Mendez‐Figueroa H , Maggio L , et al. Prevention and management of postpartum hemorrhage: a comparison of 4 national guidelines. Am J Obstet Gynecol. 2015;213(1):76.e1‐76.e10. 10.1016/j.ajog.2015.02.023 25731692

[2] Gibbins KJ , Weber T , Holmgren CM , Porter TF , Varner MW , Manuck TA . Maternal and fetal morbidity associated with uterine rupture of the unscarred uterus. Am J Obstet Gynecol. 2015;213(3):382.e1‐382.e6. 10.1016/j.ajog.2015.05.048 26026917

[3] Conrad LB , Groome LJ , Black DR . Management of persistent postpartum hemorrhage caused by inner myometrial lacerations. Obstet Gynecol. 2015;126(2):266‐269. 25923024 10.1097/AOG.0000000000000757

[4] Zwart JJ , Richters JM , Ory F , de Vries JI , Bloemenkamp KW , van Roosmalen J . Uterine rupture in The Netherlands: a nationwide population‐based cohort study. BJOG. 2009;116(8):1069‐1078; discussion 78–80. 19515148 10.1111/j.1471-0528.2009.02136.x

[5] Hayashi M , Mori Y , Nogami K , Takagi Y , Yaoi M , Ohkura T . A hypothesis to explain the occurence of inner myometrial laceration causing massive postpartum hemorrhage. Acta Obstet Gynecol Scand. 2000;79(2):99‐106. 10696956 10.1034/j.1600-0412.2000.079002099.x

[6] Buhimschi CS , Buhimschi IA , Malinow AM , Kopelman JN , Weiner CP . The effect of fundal pressure manoeuvre on intrauterine pressure in the second stage of labour. BJOG. 2002;109(5):520‐526. 12066941 10.1111/j.1471-0528.2002.01399.x

[7] Sturzenegger K , Schäffer L , Zimmermann R , Haslinger C . Risk factors of uterine rupture with a special interest to uterine fundal pressure. J Perinat Med. 2017;45(3):309‐313. 27235667 10.1515/jpm-2016-0023

[8] Kuijsters NPM , Methorst WG , Kortenhorst MSQ , Rabotti C , Mischi M , Schoot BC . Uterine peristalsis and fertility: current knowledge and future perspectives: a review and meta‐analysis. Reprod Biomed Online. 2017;35(1):50‐71. 28456372 10.1016/j.rbmo.2017.03.019

[9] Hanuman S , Pande G , Nune M . Current status and challenges in uterine myometrial tissue engineering. Bioengineered. 2023;14(1):2251847. 37665570 10.1080/21655979.2023.2251847 PMC10478746

[10] Kaplanoglu M , Kaplanoglu D , Bulbul M , Dilbaz B . Inner myometrial laceration ‐ an unusual presentation of antepartum and postpartum hemorrhage: case reports and review of the literature. J Matern Fetal Neonatal Med. 2016;29(16):2621‐2624. 26456511 10.3109/14767058.2015.1094795

[11] Abu‐Rustum RS , Abu‐Rustum SE , Abdo BK , Jamal MH . Inner myometrial laceration causing a massive postpartum hemorrhage: a case report. J Reprod Med. 2006;51(2):135‐137. 16572915

[12] Inner myometrial laceration: a newer entity causing postpartum hemorrhage. J Gynecol Surg. 2012;28(6):439‐440.

